# Understanding place: Aboriginal ways of connecting to place – a critical literature review

**DOI:** 10.1080/00049530.2026.2630451

**Published:** 2026-03-01

**Authors:** Taneisha Webster, Laura Jobson, Cammi Murrup-Stewart

**Affiliations:** 1 School of Psychological Sciences, Monash University, Clayton, Australia; 2 Gukwonderuk Indigenous Health Workforces Centre, Monash University, Clayton, Australia

**Keywords:** Aboriginal, belonging, place attachment, place connection, place identity

## Abstract

**Objective:**

The objective of this critical literature review was to understand from an Aboriginal experience what is place, how do place attachments form and their wellbeing impacts. Little is known about Aboriginal people’s place attachments in Australia. Understanding how and why Aboriginal people form place attachment has implications for health, wellbeing and policy.

**Method:**

This review was conducted in January 2025 exploring place attachments for First Nations people worldwide. Studies looking specifically at connection to Country were excluded.

**Results:**

The review identified seven articles and one poem exploring First Nations people’s understanding and experiences of place. The Australian studies were conducted in Victoria and New South Wales, whilst the international study was conducted in Canada. Including both short-term and long-term studies of connection to place demonstrated that place was underpinned by culture and had positive wellbeing implications.

**Conclusions:**

Place attachment was influenced by relational experiences (socially, spiritually/ancestrally, with Country and animals), the physical environment (decolonised, cultured) and opportunities to actively participate (learn, share, practice skills and engage in ceremony). Place was imbued with meaning, relationships and safety and through engagement with others (human and non-human), Country and culture people developed a powerful connection.

## KEY POINTS

**What is already known about this topic:**

1. Place-based attachment occurs when people create meaningful connections to spaces, often described as cognitive-emotional bonds, and is observed across cultures, place types and eras.
2. It is well established in the field of psychology that place-based attachments have positive social, emotional and psychological benefits (i.e. sense of belonging, memory support, ancestral and cultural connection and stress reduction), improving overall life satisfaction and quality, and increasing social capital, and eco-restoration responsibilities. Removal of these attachments can have negative impacts for wellbeing.
3. There is limited understanding and research from an Aboriginal perspective about place, place-based attachments and their benefits.

**What this study adds:**

1. This article explores the concept of *place* for Aboriginal people and how place-based attachments are created and prioritises Aboriginal ontology and epistemology.
2. This article details characteristics which influence Aboriginal people’s development of place-based attachment and lays the foundation for a cultural place-based framework.
3. This article highlights the potential for Aboriginal health and wellbeing to be improved through place-based connections and for further research exploring the specific characteristics of place and their psychological benefit.

Place is at the core of our epistemology and ontology, particularly for Aboriginal peoples, as it describes our history and where meaning arises, it details where we are and etches who we are (Cresswell, 2009). Place is a living thing, a space for deep connection, of belonging and reciprocity; “*place attracts us, calls us to connect and to care”* (Rey, 2021, p. 2). As Larsen and Johnson (2017) show, place creates, speaks and teaches, it orients us to our interconnectedness with others in the relationships of creation; our being-together-in-place. Place is more than its material makeup, it is engraved with interactions, and inscribed on the story of people, both in the sense of point of reference and of impact or influence (Cresswell, 2009). Place, as an Aboriginal concept, implies that there is no division between the observing mind and anything else; there is no external world to inhabit (Cresswell, 2009; Graham, 2009). Place is part of us, just as we are part of it.

Defining place is a complex task, one which requires both abstract and logical thought. Place is not universal in its meaning or effects, whilst common relational themes underpin the creation and maintenance of place, it is experiences, valued, and interpreted in culturally, historically, and individually specific ways. Understanding place from a cultural and historical context is important for Aboriginal people as this can influence how it is used, seen or experienced (Kingsley et al., 2021). Place meanings and values are created and maintained through social engagement, both individually and collectively, and it is through the practice of engagement that a sense of belonging is developed (Poe et al., 2016). As Ingold (2000) notes “a place owes its character to the experiencesd it affords to those who spend time there … it is from this relational context of people’s engagement … that each place draws its unique significance” (p. 192). Place epitomises the human story beyond the physical/seen world. As Graham (2009) states “Place underpins inquiry in the deepest ontological sense … it is the fundamental existential quantifier; it informs us of where we are at any time, thereby at the same time informing us of who we are” (p. 4). Without place, people are disconnected.

Many studies have explored how people develop a connection to specific places and the values attributed to them, which are influential in supporting health and wellbeing. Place attachment is described as a positive bond between a person and a place, where affect, cognition and behaviour are influenced by place and a desire for closeness to place occurs (Hernández et al., 2021; Hidalgo & Hernandez, 2001; Scannell & Gifford, 2010). Place attachments bring about purpose, memories, social activity and relationships, history and culture to people (Scannell & Gifford, 2017). People participate in place-making, the memorable events and activities which they partake in are key to developing meaningful attachments to place (Cresswell, 2009). It is through these social engagements that we create connections to place, which is more than its physical characteristics (Poe et al., 2016). An attachment to a certain place helps develop a community’s engagement with it, and leads to repeat visitation, environmental stewardship and ecologically sustainable behaviour (Gosling & Williams, 2010; Inglis et al., 2008; Nisbet & Zelenski, 2013). Connection to place is important for individual and collective wellbeing. It fosters past, present and future experiences (memories, goals and aspirations), whilst also strengthening responsibility to care for and protect Country.

Social and emotional wellbeing is deeply influenced by our ability to connect, belong and have purpose. It is recognised that we must connect to places, to build a sense of belonging, to care for Country and partake in society (Rey, 2021). Poe et al. (2016) purport that engaging in multiple place-making experiences enables strong connections to develop with place, creating a history, which is interconnected with the who, why and where. It is the lasting story created with place which underpins the positive effects of place and place attachment. Whilst these connections to place vary across cultures, regions and place types, it is clear that such a connection has value. Despite the breadth of research on place attachment, there is little evidence detailing Australian First Nations Peoples’ attachment to place.

Commonly, within the context of Aboriginal wellbeing, place is associated with Country. It is well established that Aboriginal Peoples’ connection to Country is central to wellbeing (Dudgeon et al., 2014; Gee et al., 2014). Country is part of Aboriginal People just as Aboriginal People are part of Country, an intrinsic connection greater than land, sea and sky (Taylor-Bragge et al., 2021). Attachment to Country runs deep within, through generations of Aboriginal families back to creation itself. The importance of such a profound connection cannot be separated from Aboriginal wellbeing research. Country is a place of belonging and a way of seeing and interacting with the world (Terare & Rawsthorne, 2020). Through connecting to Country, and by extension culture, community and ancestry, Aboriginal people are caring for their wellbeing and that of their kin and community (Dudgeon et al., 2014; Garvey et al., 2021). When this connection is lost, ruptured or difficult to access, it has devastating consequences for health and wellbeing (Gee et al., 2014). It is through this belonging (to Country, family and culture) that Aboriginal people have thrived for thousands of years.

Whilst Country, and the connection Aboriginal people have to Country, is a key tenet of social and emotional wellbeing (Dudgeon et al., 2014; Garvey et al., 2021; Gee et al., 2014), there is need to explore the broader conceptualisation of place. It is important to understand how connections to place that are not necessarily one’s ancestral or traditional Country are formed, especially given the impacts of colonisation, displacement, Stolen Generations and urbanisation and with many Aboriginal people living in metropolitan areas understanding how place may impact social and emotional wellbeing is important. Place is commonly used interchangeably with Country, though we posit that there is a difference that demands interrogation. While a clear dichotomy may not be entirely achievable, particularly in lived experience, analytic slippage between Place and Country risks obscuring their distinct ontological, cultural and political dimensions. By understanding how place-making occurs and the circumstances which enable these connections to form, improvements to health and wellbeing, policy and community organisations can be achieved.

The aim of this review, therefore, was to examine the connections Aboriginal communities have to places of significance, and how these connections foster a sense of place attachment that may reflect or emulate their connection to Country. Furthermore, the review will explore the social and emotional wellbeing benefits and healing for Aboriginal people who engage in community built places. Knowledge about connection to place for Aboriginal people can become part of health and wellbeing goals and strategies and have significant implications for policymaking and may foreground a different way to address health and wellbeing inequities. This critical literature review, therefore, aimed to capture how (a) Aboriginal people define place, (b) these relationships with place are formed and (c) connection to place influences wellbeing. Additionally, given theoretically (Gee et al., 2014) and empirically (Taylor-Bragge et al., 2021) connection to Country has positive wellbeing impacts for Aboriginal people, it is anticipated that place should also have positive wellbeing impacts for Aboriginal people.

## Method

### Methodological approach

The critical literature review employed an Indigenous research paradigm (Wilson, 2008), led by an Aboriginal PhD candidate. A critical literature review approach was used to address the research questions. The review is a detailed analysis and assessment of relevant literature to generate theory and identify research gaps. Following the three steps of a critical literature review, research was identified and obtained (assembling), organised and summarised (arranging), and evaluated to identify theory and research opportunities (assessing) (Fernandez, 2019).

### Positionality statement

This paper represents the first publication arising from Taneisha Webster’s doctoral research. Taneisha Webster is a Wathaurong woman and PhD candidate whose lived experience, community connections and professional background inform her interest in understanding Place, distinct from Country, as a determinant of Aboriginal wellbeing and as a potential site of healing. Professor Laura Jobson is a non-Indigenous woman, a settler clinical psychologist with extensive experience in trauma, culture and mental health research. She is a doctoral supervisor on this project, approaching the work as an ally committed to reflexivity, accountability and continual learning. Associate Professor Cammi Murrup-Stewart is an Aboriginal woman with family experience of the Stolen Generations and expertise in First Nations wellbeing, sociology, psychology education and Indigenous research methodologies. She is the senior doctoral supervisor on this project. Together, the authors bring complementary standpoints that shape the conceptual framing, interpretation and ethical commitments of this work.

### Search strategy

Preliminary searches were conducted to develop key search terms and eligibility parameters to ensure appropriate and adequate research data would be included in the review. A specific focus of these searches was determining the correct terminology, a vital step when acknowledging the language differences between academic literature and Aboriginal community. Pilot searches revealed limited potential of collecting research data relating to Aboriginal People in Australia, thus a broader criterion which included all First Nations People worldwide was agreed upon.

A range of electronic databases and “grey” literature resources were identified to ensure a broad range of literature was included in the review. In addition, literature known to the researcher, colleagues and Aboriginal community was included in the search. The search included four key terms paired with Aboriginal and Indigenous; place attachment, place connection, place identity and belonging. These were applied to each of the following:

- *Academic research databases*: PubMed, Scopus, Ovid and Informit.
- *Grey literature databases*: Australian Health InfoNet and Lowitja Institute.

### Inclusion and exclusion criteria

The review included materials relating to “First Nations People” (also referred to as “Indigenous”, “Native” and “Aboriginal peoples” in various regions) worldwide and “place”.

As the purpose of the review aimed to fill the gap that exists within literature regarding the broader conceptualisation of place, the review excluded studies investigating connection to Country. This decision was methodological rather than hierarchical and reflects the analytic focus of the review, rather than the relative significance of Country. At the same time, the review acknowledges the conceptual and lived overlap between Country and Place. Accordingly, studies were included where participants were engaging with place-based meanings and attachments, even where some participants were also Traditional Owners, recognising that people’s experiences of Place are not neatly separable from their cultural, historical and relational contexts. Whilst Country, the land in which Aboriginal people and their ancestors belong to, place of creation, dreaming and songlines, and where culture, tradition and lore comes, is a fundamental component of Aboriginal culture, this review looked at people’s conceptualisation and attachment to *any* place. Therefore, including the extensive connection to Country literature would have skewed the results. However, the understanding of connection to Country does still inform the interpretation of the literature and how similarities between the two could be utilised to benefit Aboriginal people’s wellbeing. The review also excluded research looking at how living in a particular geographical location influenced one’s health and wellbeing.

### Synthesis and interpretation

The remaining full texts were retrieved and read by the first author, to further assess their eligibility for inclusion. The first author conducted the review, screening all titles and abstracts against the inclusion criteria. Analysis was led by the first author, with an Indigenous senior author and non-Indigenous secondary author engaging in collaborative yarning (Shay, 2019) to ensure cultural and methodological integrity.

## Results

### Overview of search results

A total of 540 separate texts were identified, and their titles and/or abstracts screened using the inclusion and exclusion criteria. Of these, 34 were identified for possible inclusion and their full text sought. Decisions regarding inclusion were informed by ongoing consultation within the author team, with particular attention to conceptual distinction between place and Country. Subsequently, eight texts were included in the review, this included six academic research reports, one community report and one poem.

### Overview of findings

Within the literature, there was evidence to confirm Aboriginal communities have long been connecting to places purposely created by the community and which support the overall wellbeing of Aboriginal people. While there was strong evidence demonstrating the fundamental need for Country for Aboriginal people and the effectiveness of Aboriginal place-based services in addressing health needs, there was limited academic research examining the concept of place, place attachment and any associated wellbeing implications for Aboriginal people. Furthermore, while significant research has defined the notion of place-based attachments or sense of place and determined the associated social, psychological and emotional benefits for the general population, the literature reviewed highlighted that there are differences for First Nations peoples. A summary of the findings is presented in Table 1.

**Table 1.** Aboriginal place attachment.

Authors	Purpose	Geographical location	Research design	Traditional Owners or non-Traditional Owners	Participation with place	Impacts of place connection
Clapham, Senior, Longbottom, Harwood, et al. (2024a)	Explored peoples understanding, interpretation of and embodied experience of place at Jigamy Farm	Yuin Country in Pambula, NSW.	Auto-ethno-cartography/community mapping	Traditional Owners and local Aboriginal people.	Place attachment formed through repeated cultural interactions with other Aboriginal people, culture, country and knowledge.	Reconnect with culture, participate in building and having a sense of control of place/self-determination, collective pride, cultural preservation and security for the future generations.
Clapham, Senior, Longbottom, Bessarab, et al. (2024b)	Investigated the importance of place-based services for Aboriginal people in NSW.	East coast of NSW.	Collaborative process (or praxis)	Traditional Owners and local Aboriginal people.	Aboriginal concepts of place focus more on the network of connections compared to the geographical locality of place.	Builds strength and resilience for individuals and communities. Aboriginal people can become vulnerable when disruption to place occurs.
Goggin et al. (2017)	To explore how people use their physical senses, emotions and cognitive processes to create place-based attachments.	Mutti Mutthi, Ngyiampaa and Paakantji Country in Mungo National Park, NSW.	Semi-structure short interviews.	Traditional Owners and non-Indigenous (this studies analysis included Aboriginal participants only)	Physical participation, natural/striking landscape, feeling Country/culture.	Place identity, improved social/mental/spiritual wellbeing.
Kingsley et al. (2021)	Understanding the health and wellbeing benefits of Aboriginal Gathering Places in Victoria.	Victoria	Semi-structured interviews and focus groups. Two independent reference groups.	Local Aboriginal people.	Community connections, culture, safe/welcoming, learning, supportive.	Sense of belonging, pride, improved social/mental/spiritual wellbeing and cultural identity, access to supports.
Poe et al. (2016)	Investigated place based attachments in Puget Sounds, Canada.	Puget Sounds, Canada.	Mixed methods; semi-structured interviews and participatory workshops.	Indigenous Traditional Owners and non-Indigenous participants.	Multidimensional concept; 1. place making activity, 2. Cultural and familial heritage, 3. Personal and emotional experiences, and 4. Social relational connections.	Strong bonds, memories, emotions, culture.
Porter and Webster (2020)	Investigated the importance of Willum Warrain Aboriginal Gathering Place to local Aboriginal people.	Bunurong Country in Hasting, VIC.	Individual yarning and thematic/content analysis.	Local Aboriginal people.	Place is spiritual, cultural, safe, two-way relational and community focused.	Is healing, develops a sense of cultural responsibility to self, others and land, build connection and belonging.
Yashadhana et al. (2023)	To investigate how a cultural camp impacts wellbeing and connection to place among older Aboriginal people who are survivors or descendants of the Stolen Generations.	Yuwaalaraay Country, NSW.	Yarning circles, interpretive phenomenological methods.		Ceremony, speaking language, sharing traditional stories and interacting with plants, animals and artefacts.	Sense of reconnection, cultural pride, wellbeing, place attachment.

Across all eight texts, place was intrinsically linked to culture; past, present and future. The cultural foundation of place created a feeling of safety. For example, Clapham, Senior, Longbottom, Harwood, et al. (2024) described Jigamy as a powerful and safe place where people can “heal, connect, strengthen their cultural identity, and continue their cultural practices as their old people have done since time immemorial” (p. 3). Similarly, Porter and Webster (2020) described place as being “wrapped in culture” (p. 8). Place also provided the foundation to build a sense of belonging, connection and strength in culture, as expressed by a participant at Willum Warrain: “This is my mob. This is my place” Porter and Webster (2020, p. 12) and participants of gathering places across Victoria (Kingsley et al., 2021). Culture was the key aspect at “Walaay” a “ceremonial camp that occurs in cultural landscapes” at Dharriwaa (meaning “a place for all” in Yuwaalaraay language) a traditional corroboree site (Yashadhana et al., 2023, p. 3). Yashadhana et al. (2023, p. 3) reported that Walaay aimed to “reconnect to Country, intergenerational healing, reclaiming languages, and traditional ceremonies, medicine and food knowledge” while also engaging with the Traditional Owners who shared place-based cultural stories and knowledge.

Place was consistently described as holding stories, relational experience and emotional ties, with reports of improved social and emotional wellbeing and healing. Clapham, Senior, Longbottom, Bessarab, et al. (2024) described Aboriginal connection to place is “characterised by complexity, fluidity, and reflects deeply embedded stories, feelings, and emotions” (p. 8). Similarly, Clapham, Senior, Longbottom, Harwood, et al. (2024) states “Jigamy Farm is about history, identity, self-determination” (p. 8), where participants reconnect with their culture, built place and collective pride in their achievements, and fostered a sense of control, self-determination and preservation of culture and place for future generations. At Willum Warrain, participants detailed complex interconnected aspects of connection and belonging to place, echoed by Kingsley et al. (2021), highlighting the importance of these spaces for healing and wellbeing.

Aboriginal participants detailed a profound feeling of connection and healing, of being at home, belonging, and being safe (Clapham, Senior, Longbottom, Harwood, et al., 2024; Goggin et al., 2017; Kingsley et al., 2021; Porter & Webster, 2020). Place was seen to be spiritual, as participants described receiving messages from ancestors and the entity of Country, feeling welcomed and being acknowledged by Country and a sense of responsibility to Country and its cultural significance (Clapham, Senior, Longbottom, Harwood, et al., 2024; Goggin et al., 2017; Kingsley et al., 2021; Porter & Webster, 2020). While these experiences resonate with the SEWB framework’s emphasis on connection to Country, they were articulated through engagement with specific places and place-based practices, rather than through explicit claims of ancestral or ontological belonging. In this way, Place functioned as a relational site through which spirituality, responsibility and care were enacted, without necessarily equating Place with Country. Developing a sense of place connected Aboriginal people to the past, present and future, increasing wellbeing and healing (Kingsley et al., 2021). Goggin et al. (2017) reported care and play as the most mentioned emotional categories associated with place, encompassing, a sense of home, belonging, community, connection, joy, happiness and pride. These positive emotions suggest that interactive experiences fostered emotional attachment to place and connectedness to Country and enhanced social wellbeing.

Community networks and cultural ties were also critical to shaping place attachment, often more so than geographical location. As Clapham, Senior, Longbottom, Bessarab, et al. (2024) observed, “All the work we do, we done it as community, and it will always be like that” (p. 6). The complex layering of these connections to place built strength and resilience but also left Aboriginal people vulnerable when places were disrupted and damaged. Likewise, Indigenous participants in Puget Sounds reflected on the importance of deep historic ancestral ties to the land and cultural memories, and knowledge of Country being vital for connection to place (Poe et al., 2016).

Aboriginal peoples’ experience of place was influenced by the physical space. Yashadhana et al. (2023) found that physical presence “on Country”, sensory aspects of the natural environment (i.e., smells, sounds) and experiencing the uncolonised landscapes were important to participants connection to place. Findings from Goggin et al’.s (2017) study reinforced this value, with 84% of Aboriginal participants mentioning “sight” helped with connection to the past, with the striking landscape and tranquillity highly valued. Poe et al. (2016) found that physical aesthetics, sensory experiences, emotional bonds and histories of place-making activities all influenced place attachment. Porter and Webster (2020) further highlight the importance of caring for Country and regenerating land into a thriving natural space, which in turn contributed to community health.

Active participation was also critical in shaping Aboriginal people’s attachment to place. Clapham, Senior, Longbottom, Harwood, et al. (2024) reported that repeated cultural interactions with other Aboriginal people, culture, Country and knowledge fostered strong attachments at Jigamy, with great value attributed to this. Goggin et al’.s (2017) study highlighted the importance of physical participation in deepening the experience and embodying learning. Being able to be present in the space helped connection, including walking on Country and hearing messages through the wind or birds. Restoration activities also created opportunities for social connections and strong bonds to place as people joined together to achieve common goals (Poe et al., 2016). These findings demonstrated that sense of place is a multidimensional aspect of human wellbeing, often engaged in more than one place-making experience, including both material and non-material features in complex and interconnected ways. Goggin et al’.s (2017) study further quantified that interactive experiences sustained place identity and enhanced the social, mental and spiritual wellbeing of Aboriginal participants.

**Figure 1.**
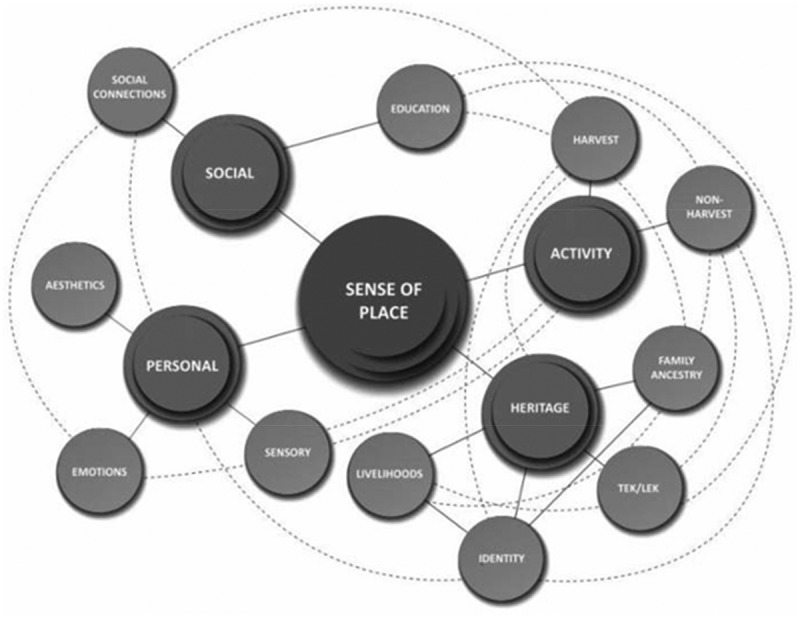
From Poe et al. (2016).

Poe et al. (2016) developed a conceptual model of place. As depicted in Figure 1, this model details four key dimensions and 11 extra elements, which highlight the interconnected and complex experience of, and attachment to, place (including its development and maintenance). The four key dimensions are as follows: (1) harvesting and other place making activities, (2) cultural and familial heritage, (3) personal and emotional experiences and (4) social relational connections.

As can be seen in the below extract from a poem written by Joanne Dywer (2014) about the Victorian Aboriginal Health Service, located on Wurundjeri Country in Fitzroy, Victoria; spaces which are created for and by Aboriginal community with the intent of supporting connection and wellbeing can create place attachment. Evident in Kingsley et al. (2021) review of Aboriginal Gathering Places which detailed vast health and wellbeing impacts for Aboriginal people and describes gathering places as “universally understood as a place of wellbeing that generated a feeling of connectedness and resilience” (p. 6). The significance and impact of Aboriginal places can span across generations.

*“Many, many years ago some elders decided*

*That their people needed a meeting place*

*Where they could come and be united …*

*Their aim was community control*

*To make decisions of their own*

*But it was more than just a meeting place*

*For many it was home … ”*

## Limitations

The analytic distinction between place and Country, while necessary to address a gap in the literature, is conceptually complex and may have resulted in the exclusion of studies where these concepts are closely intertwined in lived experience.

Expanding the scope to include First Nations peoples internationally strengthened the breadth of the review but may limit the direct applicability of findings to Aboriginal Australian contexts.

As a critical literature review grounded in an Indigenous research paradigm, the search, screening and synthesis prioritised conceptual depth and relational accountability over systematic replication, which may have influenced study selection and interpretation.

The final number of included texts was small, reflecting both the specificity of the research focus and the limited existing literature, and findings should therefore be interpreted as exploratory and theory-building rather than exhaustive.

## Discussion

Aboriginal people have always been strongly connected to Country, with an abundance of research validating the need for Country and culture for wellbeing. With the vast and complex impacts of colonisation, many Aboriginal people experience disconnection from Country (Gee et al., 2014). This review asked: how do Aboriginal people form connections to places through non-traditional avenues? What characteristics, events, experiences need to occur for a space to develop into a place of connection? And can these new connections positively impact Aboriginal people’s wellbeing?

The studies presented common themes which Aboriginal participants felt were important to feeling a sense of place and connection. These themes included being in culturally rich spaces that told stories of the past and creation, opportunities to learn knowledge and connect to Elders or knowledge holders and participation in cultural practices (i.e., building canoes and making possum skin cloaks). Aboriginal people described spiritual connections to places, where Country and ancestors spoke to them through sensory experiences such as the wind, animals or sounds of water. Such connections felt innate and if it was meant to be. Feeling safe and welcomed were also important, a space where no matter your ancestry, or depth of cultural knowledge or skills, your identity was never questioned. Many of these components are evidence in Aboriginal social and emotional wellbeing research and frameworks as key factors and determinants of health (Dudgeon et al., 2014; Garvey et al., 2021; Gee et al., 2014).

Consistent with broader literature on place attachment, this review also identified that place is more than the physical space. It is the layered connections between people, culture, history and story; a bundle of interconnected relational experiences between people, things and the metaphysical world. As highlighted by Rey (2021) and Larsen and Johnson (2017), people are responsive to place, just as place is responsive to people (two-way relational). Through relational experiences meaning and values are developed, inscribed into place and maintained through cultural practices. This extends existing psychological models of place attachment, which often emphasise individual-level emotional and cognitive bonds (Scannell & Gifford, 2010), by showing that Aboriginal attachment to place is cultural, spiritual and often collective. Therefore, place must be viewed through the relational context in which it connects to and with a person, and it is through this relationality that people develop a sense of place, of belonging and connection, and identity.

For Aboriginal people, social and emotional wellbeing is heavily influenced by culture, Country and kinship and it is through these core components that wellbeing is achieved (Gee et al., 2014). Scannell and Gifford (2017) found belonging was a common benefit of developing place attachment and this brought about feelings of homeness, love, fitting in, having ties to a place and connecting with others. Active participation, whether through ceremony, learning traditional practices and stories, or building relationships with others, was central to attachment, underscoring that place attachment is not passive, but created through “doing in place” (Cresswell, 2009). As Yashadhana’s et al. (2023) state, cultural experiences, like Walaay, are “powerful downstream, grassroots initiatives for improving the health and wellbeing of Aboriginal people” (p. 6). Therefore, by developing a connection and sense of belonging through an Aboriginal place that is culturally strong, spiritual, relational and safe would create greater opportunities for healing and create stronger futures for individuals and their families. These findings demonstrate that Aboriginal place attachment can be understood as a multidimensional psychological process, producing wellbeing through cultural, social, emotional and spiritual engagement.

These findings highlight important implications for psychology and the social and emotional wellbeing of Aboriginal communities. Aboriginal spaces such as Jigamy and Willum Warrain are embedded within Aboriginal culture and provide safe spaces to learn, connect and belong. Aboriginal gathering places in Victoria also demonstrated how engagement with cultural spaces increased access to health services and information and increased access to support. As shown in the review, places which are intrinsically cultural enable Aboriginal people to engage in processes of learning, healing and belonging, supporting the development of long-term relationships with place.

Therefore, formalised Aboriginal spaces hold key characteristics that support place attachments and have a cultural and social responsibility to maintain these defining features for their communities. Strengthening culturally embedded place attachments represents an important pathway through which Aboriginal community wellbeing can be supported. This should be reflected in policy and practice with Aboriginal organisations receiving adequate funding and support to create culturally strong places which are self-determining and prioritise Aboriginal ontologies and epistemologies.

For psychologists, these places should be recognised as protective factors for wellbeing. Psychological practice, including intervention design and community-based work should consider how engagement with culturally strong spaces and programmes can be meaningfully integrated. Whilst more research is needed, these findings lay the foundations of what “place” looks like for Aboriginal people within contemporary health and wellbeing contexts.

The evidence in the review highlighted key dimensions of Aboriginal place attachment:

1. For a space to become important, and for a sense of connection and belonging to be made, it must hold meaning developed through lived experience. As Cresswell (2009) notes, spaces only become places when they are imbued with values and significance, a process Rey (2021) describes as storying. For Aboriginal people, this meaning arises through culture, Country and community. When places are embedded in these contexts, attachment is more likely to occur, promoting positive wellbeing outcomes including belonging, identity, pride, social connection, spirituality and healing. These outcomes align with established Aboriginal social and emotional wellbeing frameworks (Dudgeon et al., 2025; Garvey et al., 2021; Gee et al., 2014).
2. The environment itself was central to connection. Places with cultural significance, uncolonized or natural landscapes, and sensory richness (sounds, smells and sights) created depth of experience and opportunities for relational engagement, such as feeling the presence of Ancestors. These environments also support learning of history and culture, leading to increased pride, belonging and social connection. As Terare and Rawsthorne (2020) note, Aboriginal people belong and understand through Country, making the qualities of place critical for attachment. Place-making activities further reinforced this dimension, instilling responsibility to care for Country while also fostering ecological, cultural and social wellbeing. This echoes broader findings that strong place attachment encourages environmental stewardship (Gosling & Williams, 2010; Inglis et al., 2008; Nisbet & Zelenski, 2013).
3. Active participation was central to developing place attachment. Being present and engaged, through ceremony, cultural practice, learning or restoration, fostered pride, embodied learning and responsibility to place. As Cresswell (2009) notes, “places are practices”; meaning arises from doing. Across the reviewed studies, the relational nature of activities (emotional, social, intellectual, spiritual and physical) was more important than the specific activity itself, with meaning and belonging emerging through lived experiences (Ingold, 2000; Poe et al., 2016).

The permanency and accessibility of spaces supported stronger and more enduring attachments, enabling cultural activities to accumulate across time. Such ongoing participation strengthened cultural knowledge and skills, built community networks and created intergenerational connections, all of which acted as protective factors for wellbeing. Examples such as Jigamy and Willum Warrain show how Aboriginal gathering places cultivate pride, cultural continuity and belonging not only for individuals but also for families and future generations.

Even short-term engagements, such as cultural camps, produced profound psychological and emotional impacts, echoing broader cultural research (e.g., Gilby et al., 2022). These findings raise questions about how different forms of attachment – temporary versus long term – contribute to wellbeing and highlight the need for further research to explore these complexities. Future work should also aim to capture the emotional and spiritual dimensions of Aboriginal place attachment more precisely, including through the development of culturally appropriate measurement tools, as current psychological scales often overlook these central aspects (Goggin et al., 2017).

Whilst the academic evidence is sparse, the studies offered powerful descriptions of how place, connection and belonging to place promote wellbeing. Developing a comprehensive framework of Aboriginal place and place connection would support the growth and development of spaces, programs, practices and organisations, where Aboriginal people gather and build attachments and improve wellbeing. Greater understanding of these processes would also inform policy by supporting investment in culturally embedded, accessible, enduring (intergenerational) and safe places as a strength-based preventative strategy to improve wellbeing.

## Conclusion

This review was designed to understand (1) how Aboriginal people conceptualise place, (2) what characteristics or experiences occur to form place attachment and (3) if the development of place attachments influences wellbeing. Our findings suggest that place is not simply a space but is a bundle of networks of relational experiences which people have. These experiences are what develop meaning and help people to form attachments to place. For Aboriginal people “place” must be embedded within culture; a space which is culturally strong and safe, where people feel welcomed and accepted for who they are and can build relationships with land, community and knowledge. Positive connections to place which develop strong meanings, are two-way in nature, have long-term goals, strengthen culture and social networks and prioritise Aboriginal ways of being, which in turn all influence health and wellbeing.

The process underlying the development of place and place-based attachments constitutes a complex, interrelated framework that requires careful examination to enhance understanding. The implications of this could support improvements in intergenerational challenges, break trauma cycles and disconnection, to establish strong foundations for generations to come and increase community-wide wellbeing. Exploration of how a “place” is formed would increase our understanding of how to develop and build more places where Aboriginal people can connect and promote social and emotional wellbeing and lead to greater advances to closing the gap and health policy.

## Data Availability

The authors confirm that the data supporting the findings of this study are available within the article.
